# Evaluation of environmental Mucorales contamination in and around the residence of COVID-19-associated mucormycosis patients

**DOI:** 10.3389/fcimb.2022.953750

**Published:** 2022-09-02

**Authors:** Anup K. Ghosh, Ravinder Singh, Snigdha Reddy, Shreya Singh, Shivaprakash M. Rudramurthy, Harsimran Kaur, Hansraj Choudhary, Arunaloke Chakrabarti

**Affiliations:** Department of Medical Microbiology, Post Graduate Institute of Medical Education and Research, Chandigarh, India

**Keywords:** COVID-19 associated mucormycosis (CAM), environmental mucorales, air sampling, amplified fragment length polymorphism (AFLP), genetic relatedness

## Abstract

**Introduction:**

Recently, India witnessed an unprecedented surge of coronavirus disease 2019 (COVID-19)-associated mucormycosis (CAM) cases. In addition to patient management issues, environmental Mucorales contamination possibly contributed to the outbreak. A recent study evaluated environment contamination by Mucorales in the hospital setting. However, a considerable number of CAM patients were never admitted to a hospital before the development of the disease. The present study, therefore, planned to evaluate Mucorales contamination of patients’ residences.

**Methods:**

The residential environment of 25 patients with CAM living in north India was surveyed. Air samples were collected from indoor and immediate outdoor vicinity of the patients’ residence and cultured on Dichloran Rose–Bengal Chloramphenicol (DRBC) agar with benomyl for selective isolation of Mucorales. Surface swab samples were also collected from the air coolers fitted in those residences and cultured on DRBC agar. The isolates were identified by phenotypic and genotypic methods. Amplified fragment length polymorphism (AFLP) was employed to evaluate the genetic relatedness of the environmental and patients’ clinical isolates.

**Results:**

The median spore count (mean ± SD, cfu/m^3^) of Mucorales in the air of patients’ bedrooms was significantly higher than in the air in other rooms in those residences (3.55 versus 1.5, p = 0.003) or the air collected directly from the front of the air cooler (p < 0.0001). The Mucorales spore count in the environment did not correlate with either ventilation of the room or hygiene level of the patients’ residences. *Rhizopus arrhizus* was isolated from the environment of all patients’ residences (n = 25); other Mucorales species isolated were *Cunninghamella bertholletiae* (n = 14), *Rhizopus microsporus* (n = 6), *Rhizopus delemar* (n = 6), *Syncephalastrum racemosum* (n = 1), *Lichtheimia corymbifera* (n = 1), and *Mucor racemosus* (n = 1). Genetic relatedness was observed between 11 environmental isolates from the patients’ bedrooms and respective clinical isolates from patients.

**Discussion:**

The study supported the view that the patients might have acquired Mucorales from the home environment during the post-COVID-19 convalescence period. Universal masking at home during patients’ convalescence period and environmental decontamination could minimize exposure in those susceptible patients.

## Introduction

The coronavirus disease 2019 (COVID-19) pandemic has affected millions globally, crippling healthcare infrastructure and causing substantial socioeconomic impact worldwide. A parallel rise in concomitant bacterial and fungal infections was also seen among those patients (Lansbury et al., 2020; Segrelles-Calvo et al., 2021). However, the surge of COVID-19-associated mucormycosis (CAM) cases was unprecedented, particularly in India, from April to August 2021, prompting the Government of India to declare CAM as a notifiable disease (Segrelles-Calvo et al., 2021).

Studies evaluating the host-related risk factors associated with CAM have revealed that mucormycosis can occur among COVID-19 patients due to uncontrolled hyperglycemia, widespread and injudicious use of corticosteroids and broad-spectrum antibiotics, and use of invasive ventilation (Mishra et al., 2021; Pakdel et al., 2021; Prakash et al., 2021). While tracing the epidemiological triad, the role of the environment in the causation of the outbreak was not properly evaluated, although myriad hypotheses on environmental issues have been proposed. Mucorales species have ubiquitous distribution, and the presence of high Mucorales spore counts in the hospital and its vicinity has been reported during pre-pandemic and pandemic periods in India (Prakash et al., 2016; Prakash et al., 2020; Biswal et al., 2022). A multicenter study from India conducted during the CAM outbreak reported high Mucorales spore count in the hospital air and Mucorales contamination of air-conditioning ducts of the hospital (Biswal et al., 2022). However, we observed that a large number of patients were acquiring CAM during the post-COVID-19 convalescence period at home. Hence, in the present study, we conducted an environmental survey for Mucorales in the residential environment of CAM patients.

## Materials and methods

We approached 469 patients treated at the Postgraduate Institute of Medical Education and Research (PGIMER), Chandigarh, for CAM from 1 May to 31 July 2021 for environmental sampling of their residence, and only 25 patients consented to participate in the study. All those 25 patients acquired mucormycosis while convalescing at home post-COVID-19. The status of ventilation and hygiene/cleanliness was noted at each patient’s residence and arbitrarily categorized as good or poor based on a predefined questionnaire (**Supplementary Figure 1**). Ethical approval from the Institute’s ethical committee was taken prior to the commencement of the study.

### Sample collection and processing

#### Air sampling

Air sampler (AES Laboratoire Sampl’Air Pro sieve sampler, Hazelwood, MO, USA) with a suction rate of 100 L/min was used to collect 1,000 L of air (10 min per sample/plate) for each location using a 90-mm-diameter Petri plate containing Dichloran Rose–Bengal Chloramphenicol (DRBC) and benomyl (12 µg/ml). From each patient’s residence, we collected samples from four corners of the bedroom, living room, and immediate vicinity outside the patient’s residence. Wherever an air cooler was found attached to the bedroom, an air sample was also collected immediately from the front of the cooler after switching it “on” at the speed and position usually used by the occupants.

The labeled plates were then sealed with parafilm and transferred to the laboratory in sealed plastic zipper bags. The plates were incubated at 25°C for 7 days and inspected for growth of Mucorales once daily. Fresh colonies appearing cottony resembling Mucorales were immediately subcultured on Sabouraud dextrose agar to avoid merging the colonies growing on the same plate. The spore count data were recorded for each of the broad categories of the sampled residential area as described previously (Prakash et al., 2020). Briefly, the air spore count was obtained using the formula *N/V*, where *N* is the probable number of viable impacted particles, calculated using the number of holes in the sampling head and the numerated colonies on culture, and *V* is the controlled volume, calculated using the sampling duration.

#### Surface swabs

In-house prepared sterile normal saline-moistened cotton swabs were used to collect samples from bed railing, table, shelf, door handles, window pane, cooler floor, nebulizer, or any other material available in each patient’s bedroom. The swabs were plated onto a DRBC–benomyl plate and incubated at 25°C for 7 days as mentioned above.

### Phenotypic and genotypic identification

Colonies appearing as white or dark cottony growth were counted after checking microscopically for aseptate hyphae. The Mucorales colonies were subcultured on Sabouraud dextrose agar (SDA) and identified microscopically following standard technique (Hoffmann et al., 2013; Prakash et al., 2016). The identification of Mucorales was further confirmed by DNA sequencing using the primer pairs ITS 4 (internal transcribed spacer) (5′-TCCTCCGCTTATTGATATGC-3′) and ITS5 (5′-GGAAGTAAAAGTCGTAACAAGG-3′) (Prakash et al., 2016). The identification of clinical isolates of those patients was reconfirmed by methods as described above; in seven patients where culture failed to grow Mucorales from clinical samples although direct microscopy was positive for aseptate hyphae, the identification of Mucorales was performed by extraction of DNA from samples, amplification by the abovementioned primers and/or Mucorales-specific primers, and finally by sequencing (Prakash et al., 2016).

### Amplified fragment length polymorphism

Molecular typing by amplified fragment length polymorphism (AFLP) was performed to analyze the genetic relatedness of the environmental isolates and clinical isolates as described previously (Prakash et al., 2016). Briefly, 50 ng of DNA was subjected to restriction ligation with enzymes *Eco*RI and *Hin*dIII, 50 pmol of the respective adapters, and 1U of T4 DNA ligase, making the total reaction volume 20 µl, and incubated at 37°C for 2 h. The restricted DNA fragments were amplified by PCR, and the amplified samples were analyzed by capillary electrophoresis in the Genetic Analyzer (3500 Dx, Applied Biosystems, Foster City, CA, USA). The data were exported and analyzed using BioNumerics v6.6 software (Applied Maths, Ghent, Belgium). Only the bands ranging in size between 140 and 360 base pairs were analyzed. The genetic diversity was calculated using Pearson’s correlation coefficient, and the isolates were clustered using the unweighted pair group method with arithmetic mean.

### Statistical analysis

The data were analyzed using GraphPad Prism (version 9.0). All data are reported as frequencies and proportions or means and standard deviation (SD), as appropriate. The chi-square test and Fisher’s exact test were used to compare categorical variables. The Shapiro–Wilk test was used to check for normality of data distribution, the Mann–Whitney U test for comparison of median values case data was not distributed normally, and Student’s t-test was used to compare normally distributed data. A p-value <0.05 was considered significant.

## Results

**Figure 1:**
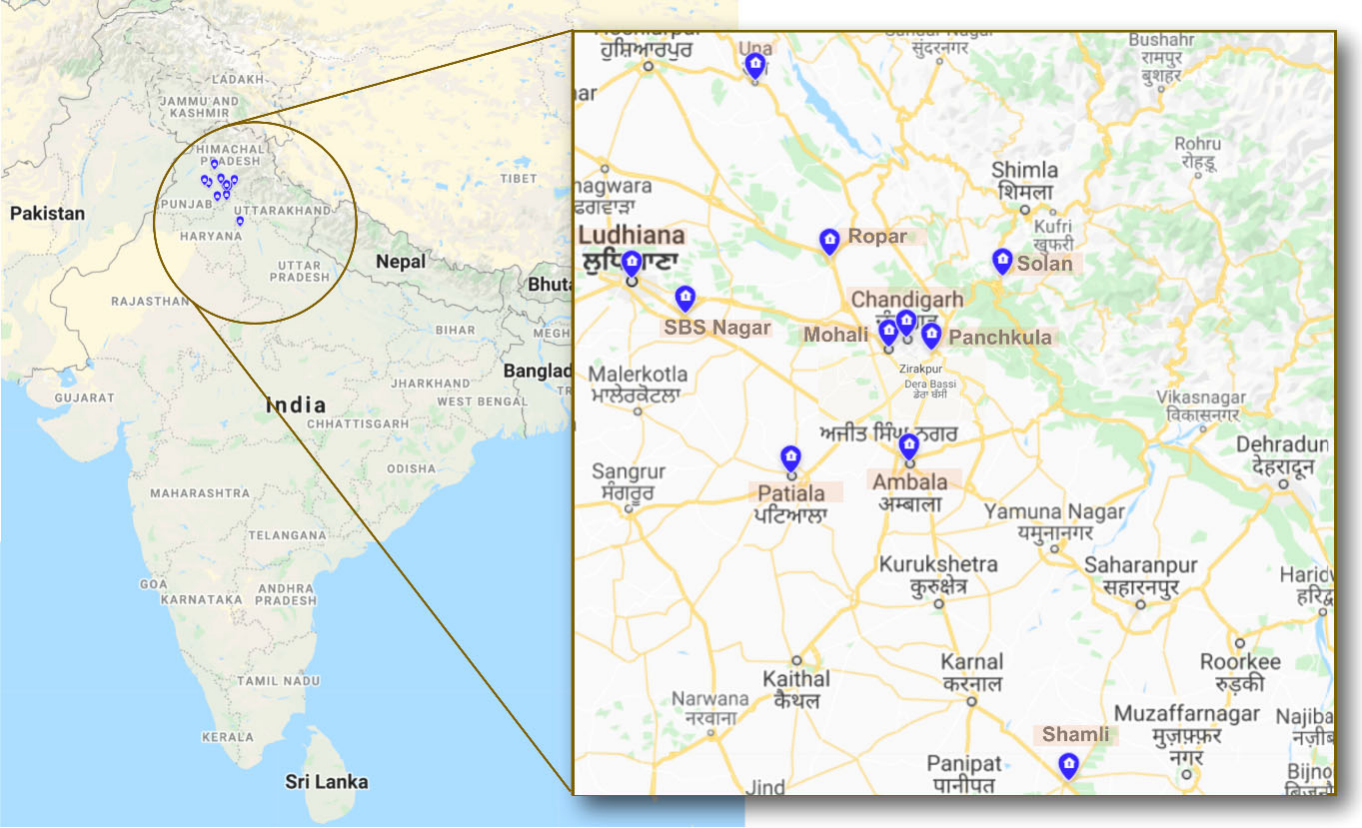
The map depicting the districts where environmental sampling was performed. The states from where sampling was performed are indicated in the circle, i.e., Punjab, Haryana, Uttar Pradesh, Himachal Pradesh, and Chandigarh. The blue highlights indicate the various districts within these states from where the samples were collected.

Among enrolled 25 patients, the majority (n = 23) presented with rhino-orbital mucormycosis; one patient had pulmonary mucormycosis, and another had gastrointestinal mucormycosis. The patients reside in five provinces/union territory, viz., Punjab (n = 13), Haryana (n = 4), Uttar Pradesh (n = 3), Himachal Pradesh (n = 3), and Chandigarh (n = 2) (**Figure 1**).

**Figure 2:**
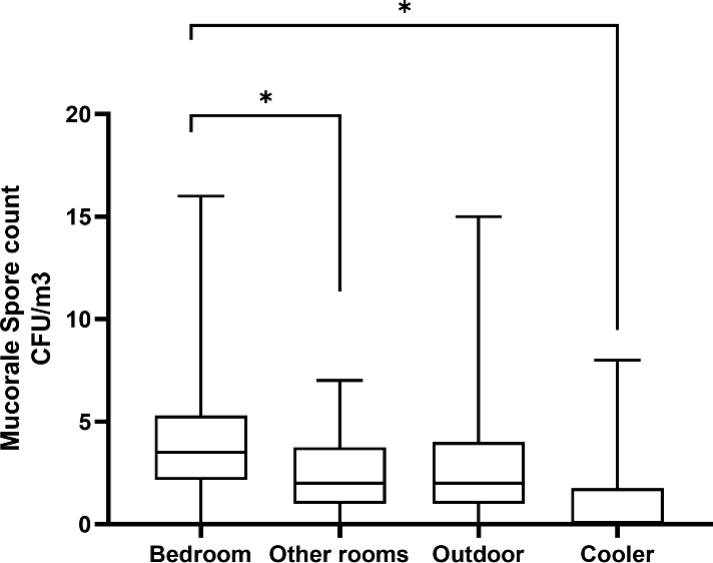
Comparison of Mucorales spore counts based on samples from the air of the residential environment. *p-value <0.05.

*Rhizopus arrhizus* was isolated from the air of all (n = 25) patients’ houses sampled. Other Mucorales species isolated from the environment include *Cunninghamella bertholletiae* (n = 14), *Rhizopus microsporus* (n = 6), *Rhizopus delemar* (n = 6), *Syncephalastrum racemosum* (n = 1), *Lichtheimia corymbifera* (n = 1), and *Mucor racemosus* (n = 1). Mucorales species isolated from clinical samples of those patients (n = 18) were *R. arrhizus* (n = 12), *R. delemar* (n = 3, differentiated from *R. arrhizus* after ITS sequencing), *R. microsporus* (n = 2), and *Rhizopus homothallicus* (n = 1). PCR sequencing directly from clinical specimens from culture-negative samples identified the implicated Mucorales species as *R. arrhizus* (n = 4), *R. homothallicus* (n = 2), and *Saksenaea vasiformis* (n = 1). In 17 out of 25 (68%) cases, the same species of Mucorales were isolated from clinical samples and the environment of the residence of the respective patients, *R. arrhizus* (n = 16, i.e., 12 culture positive and four diagnosed by PCR sequencing), and one each of *R. microsporus* and *R. delemar*. The details of data including spore count are shown in **Table 1**. Among air samples, the proportion of samples positive for Mucorales was 92% (n = 23) from bedroom air samples, followed by 68% (n = 17) from other rooms, 20% (n = 5) from cooler air, and 88% (n = 22) from outdoor air samples. The mean spore counts of Mucorales in the air are shown in **Figure 2**. The mean spore counts (mean ± SD) in air samples from the bedroom, other rooms, cooler, and outdoors were 4.4 ± 3.6, 2.6 ± 2.1, 1.3 ± 2.4, and 3.38 ± 3.3 cfu/m^3^, respectively. The median spore counts in the bedroom, other rooms, water cooler, and outdoors were 3.55 (range, 0 to 16 cfu/m^3^), 1.5 (range, 0 to 7 cfu/m^3^), 0 (range, 0 to 8 cfu/m^3^), and 2 (range, 0 to 15 cfu/m^3^). A significantly higher spore count was noted in the bedroom vs. other rooms (p=0.003), while the air from the cooler had significantly lower spore counts than all the other sites, i.e., bedroom vs. cooler (p < 0.0001), other room vs. cooler (p = 0.009), and outdoor vs. cooler (p < 0.0001). No significant difference was observed in spore counts from bedroom vs. outdoor (p = 0.123) and other room vs. outdoor (p = 0.121). Among surface swabs, Mucorales was isolated from the bed (n = 17, 68%), air cooler (n = 10, 40%), and other surfaces (n = 23, 92%).

**Table 1:** The state-wise details of different Mucorales species isolated in environmental samples from residential areas of patients with COVID-19-associated mucormycosis and its spore counts.

State (no. of patients)	Mucorales spore counts (values in cfu/m^3^) (mean ± SD)	Organisms isolated from environment	Clinical isolates
Haryana (N = 4)	Air: 3.5 ± 0.9 to 9.5 ± 5.1	*Rhizopus arrhizus* (n = 4) *Rhizopus delemar* (n = 1) *Rhizopus microsporus* (n = 2) *Cunninghamella bertholletiae* (n = 2)	*R. arrhizus* (n = 3) *R. delemar* (n = 1)
Punjab (N = 13)	Air: 0.8 ± 1.5 to 3.6 ± 3.7	*R. arrhizus* (n = 13) *R. delemar* (n = 3) *R. microsporus* (n = 2) *C. bertholletiae* (n = 6) *Lichtheimia corymbifera* (n = 1) *Mucor racemosus* (n = 1) *Syncephalastrum racemosum* (n = 1)	*R. arrhizus* (n = 7) *R. delemar* (n = 1) *R. microsporus* (n = 2) *Rhizopus homothallicus* (n = 3)
Uttar Pradesh (N = 3)	Air: 2 ± 1 to 5.6 ± 0.5	*R. arrhizus* (n = 3) *R. delemar* (n = 2) *C. bertholletiae* (n = 2)	*R. arrhizus* (n = 2) *R. delemar* (n = 1)
Himachal Pradesh (N = 3)	Air: 0.8 ± 0.8 to 3.6 ± 3.1	*R. arrhizus* (n = 3) *R. microsporus* (n = 1) *C. bertholletiae* (n = 2)	*R. arrhizus* (n = 2) *Saksenaea vasiformis* (n = 1)
Chandigarh (N = 2)	Air: 1 ± 0 to 1.8 ± 0.2	*R. arrhizus* (n = 2) *R. microsporus* (n = 1) *C. bertholletiae* (n = 2)	*R. arrhizus* (n = 2)

The comparative Mucorales spore counts in the air of the households with good versus poor hygiene ranged from 1.93 ± 2.808 to 5.17 ± 3.71 cfu/m^3^ versus 0 to 3.55 ± 3.35 cfu/m^3^, respectively; the difference was not statistically significant.

**Figure 3:**
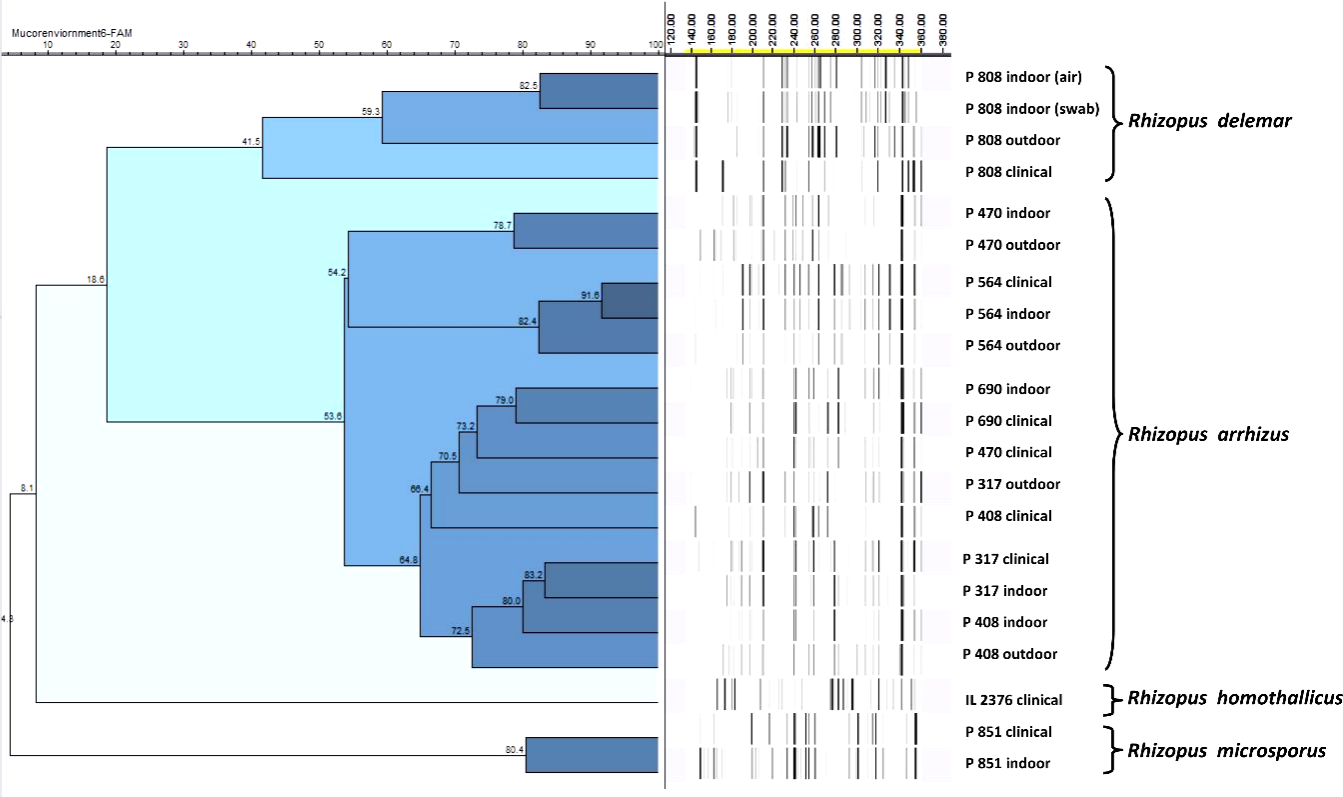
Representative amplified fragment length polymorphism (AFLP) of clinical and environmental isolates from patients and their respective residential areas. The scale bar represents the percentage similarity between the isolates.

**Figure 4:**
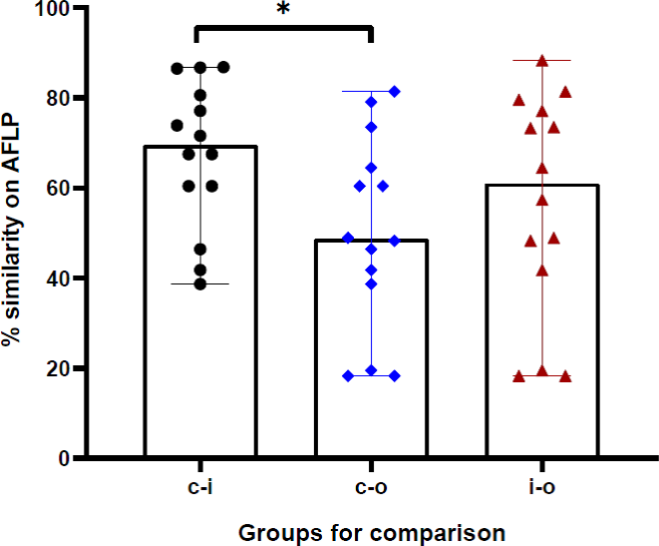
The percentage genotypic similarity based on amplified fragment length polymorphism (AFLP) between clinical and indoor isolates (c-i), clinical and outdoor isolates (c-i), and indoor and outdoor isolates (i-o) obtained from 14 cases in the study. *p-value <0.05.

The AFLP was performed when the same species that was implicated in clinical disease was also isolated from the residential environment of the respective patient. All isolates were first identified based on DNA sequencing of the ITS region. The isolates subjected to AFLP analysis included 12 *R. arrhizus* isolates recovered from clinical samples, three isolates of *R. delemar*, and two isolates of *R. microsporus*. One clinical isolate of *R. homothallicus* was also included in the AFLP. The AFLP revealed four distinct clusters, each comprising *R. delemar*, *R. arrhizus*, *R. homothallicus*, and *R. microsporus* (**Figure 3**). Genetic similarity between the clinical and corresponding indoor isolates was observed in 11 cases. All those genetically related isolates were isolated from the bedroom of the respective patient. The comparison of percentage similarity observed between clinical, indoor, and outdoor isolates is shown in **Figure 4**. A significantly higher genetic similarity was observed between clinical and indoor isolates compared to that seen between clinical and outdoor isolates.

## Discussion

In this study, we tried to evaluate the distribution of mucoralean spores in the immediate vicinity of the COVID-19 patients while convalescing at home, which may have led to the acquisition of mucormycosis. A significantly higher spore count was observed in the air samples from the patients’ bedrooms compared to other rooms. There was no association between the spore counts in the air and environmental hygiene or ventilation of the house. In the majority (68%) of the patients, the same Mucorales species were isolated from clinical samples and patients’ environments. AFLP results revealed clustering of clinical isolates and isolates from the patients’ bedrooms.

The sporangiospores released by Mucorales are ubiquitous, easily aerosolized, and readily disperse throughout the environment (Richardson, 2009). High humidity and temperature in a tropical country like India possibly provide an optimal condition for the survival and proliferation of these fungi in the environment (Chakrabarti and Singh, 2011). Although clinically relevant members of the Mucorales are found in diverse ecological spaces, the data on the burden and distribution of its spores in the air of patients’ residential areas are not well documented. Further, the contribution of environmental contamination to the causation of the outbreak is not well studied, although it is well known that uncontrolled diabetes and indiscriminate use of corticosteroids played an important role in this outbreak (Baldin and Ibrahim, 2017; Tavakolpour et al., 2020; Jose et al., 2021; Patel et al., 2021; Sabirli et al., 2021; Sen et al., 2021; Smith et al., 2021; Vlahakos et al., 2021).

The mean spore counts in air samples collected from bedrooms were significantly higher as compared to other rooms of patients with CAM and immediate outdoor environment. The Mucorales spore count in the air of the outdoor environment in the present study is similar to the spore count of the outdoor environment in an earlier study from our center (Prakash et al., 2020). Recently, a hospital-based multicenter study was conducted to investigate the role of the hospital environment in the CAM outbreak (Biswal et al., 2022). Mucorales species were isolated from 53.8% of the hospital’s outdoor air samples, 21.7% of the indoor air samples, and 11.1% of air-conditioning vents, highlighting the importance of regular surveillance and decontamination of the hospital environment.

Although lower spore counts are expected indoors compared to outdoors, less circulation of indoor air inside a closed bedroom could perhaps expose patients to higher overall spore concentrations. Additionally, during the quarantine period, patients may be too sick to clean or ventilate their rooms, compounding the spore concentrations in their rooms. The duration of exposure to air-borne spores may be another factor. Generally, patients with COVID-19 were quarantined for >2 weeks, with limited room traffic and cleaning. Non-cleaning may lead to sporulation and dispersal of Mucorales spores from dustbins with organic matters, bedding, unwashed clothes, or walls (Rainer et al., 2001). Sunlight and high humidity have been reported to restrict the dispersal of fungal spores (Norros et al., 2015). Hence, less sunlight and low humidity inside the patients’ rooms might have contributed to increased spore counts. The release of spores from the sporangium is controlled by external factors like air speed, relative humidity of the airflow, and vibration of the growth substrate (Pasanen et al., 1991; Wyatt et al., 2013). The variations in these extraneous parameters may have contributed to differences between indoor and outdoor airborne spore counts.

Although hygiene or ventilation was not significantly correlated to spore burden, the use of air purification technologies such as portable air cleaners (air purifiers or air sanitizers) and heating, ventilation, and air conditioning (HVAC) filters may minimize Mucorale spores in the homes of high-risk patients. In addition, the use of indoor humidifiers to increase indoor humidity, regular aeration of quarantine rooms, and exposure to sunlight may potentially help in reducing spore dispersal. However, in the present study, the no standard scale for assessment of hygiene and ventilation was used, also sunlight and humidity have not been quantified, which warrants further studies.

The comparison of the clinical isolate with the isolates obtained from the patients’ environment by molecular typing technique is the standard method of associating the strain with the disease. AFLP is one of the promising molecular typing techniques that have high discriminatory power. However, only limited studies have assessed the AFLP of Mucorales to describe the extent of genetic relatedness. Our AFLP results suggest high-risk patients such as those in the COVID-19 convalescence phase may acquire infection at home. Based on these findings, we suggest that patients with a high risk of acquiring mucormycosis should wear an appropriate mask even at home during the risk period. However, it is difficult to judge from our study how long one should wear a mask at home.

*R. arrhizus* was the most common Mucorales isolated from the air of the patients’ residential environment, which is in concordance with other environmental studies from India (Flannigan et al., 2011; Pathak, 2012; Roy et al., 2017; Prakash et al., 2020; Biswal et al., 2022). In the case of *S. vasiformis* infection, we were unable to recover this species from this patients’ environment probably due to less spores produced by *Saksenaea* spp. (Alvarez et al., 2010). Although the emergence of *R. homothallicus* has been recently reported in CAM (Kaur et al., 2021; Patel et al., 2021), this species produces fewer sporangiospores (Chakrabarti et al., 2010; Kokkayil et al., 2017), which could explain why we could not isolate it from the residential environment of any of the three patients infected with it. Recently, *R. homothallicus* has been recovered from environmental samples collected in hospital settings (Biswal et al., 2022). Other Mucorales like *Apophysomyces variabilis*, *Syncephalastrum* spp., *Cunninghamella* spp., and *Lichtheimia* spp. have been reported in both clinical and environmental samples (Prakash et al., 2016; Barcoto et al., 2017; Prakash et al., 2020; Singh et al., 2021). Differences in the temperature, organic substrate, sunlight, soils of potted plants, cellulosic material like cotton fabrics or books, indoor dampness, etc., probably exert influence in determining the species distribution of aero-mycobiome (Flannigan et al., 2011).

The limitations of this study may be that we did not collect samples from patients with COVID-19 from adjoining or nearby residential areas who did not develop CAM. Also, all patients in this study were diabetic, which itself predisposes to mucormycosis, and other members of their household (irrespective of diabetic status) exposed to the same environment did not develop CAM, suggesting multifactorial pathogenesis of this disease. We also did not collect samples from dampened walls or washrooms, which are other potential sites of fungal colonization. Nonetheless, this study helps to understand and elucidate the epidemiology of Mucorales causing CAM. The estimation of aero-spore burden can further help in predicting the risk of developing mucormycosis in the community setting. This also highlights the need for selective protective interventions in high-risk patient groups.

## Data availability statement

The original contributions presented in the study are included in the article/**Supplementary Material**. Further inquiries can be directed to the corresponding author.

## Author contributions

AG: study conceptualization, methodology, and data collection. RS: data collection, data analysis, and methodology. SR: data collection and methodology. SS: data analysis and manuscript writing. SMR: critical revision of manuscript and supervision. HK: critical revision of the manuscript. HC: methodology. AC: supervision, study design, and critical revision of the manuscript. All authors listed have made a substantial, direct, and intellectual contribution to the work. All authors have approved the final manuscript for publication.

## Acknowledgments

We would also like to acknowledge Mrs. Sunita Gupta, MSc, for her technical assistance.

## Conflict of interest

The authors declare that the research was conducted in the absence of any commercial or financial relationships that could be construed as a potential conflict of interest.

## Publisher’s note

All claims expressed in this article are solely those of the authors and do not necessarily represent those of their affiliated organizations, or those of the publisher, the editors and the reviewers. Any product that may be evaluated in this article, or claim that may be made by its manufacturer, is not guaranteed or endorsed by the publisher.
