# Supplementary material for: Evaluation of environmental Mucorales contamination in and around the residence of COVID-19-associated mucormycosis patients
Source: Front Cell Infect Microbiol. 2022 Sep 2;12:953750. doi: 10.3389/fcimb.2022.953750 (PMC9478190; doi:10.3389/fcimb.2022.953750)
Supplement: Supplementary file 1 [file Table_1.docx]

Supplementary Table 1: ITS region sequenced, identified and submitted to GenBank

| S. No. | Lab ID | Mucorale identified | GenBank Accession number |
| --- | --- | --- | --- |
| 1 | P-317 clinical | *Rhizopus arrhizus* | OP161818 |
| 2 | P-317 indoor | *Rhizopus arrhizus* | OP161819 |
| 3 | P-317 outdoor | *Rhizopus arrhizus* | OP161820 |
| 4 | P-408 clinical | *Rhizopus arrhizus* | OP161821 |
| 5 | P-408 indoor | *Rhizopus arrhizus* | OP161822 |
| 6 | P-408 outdoor | *Rhizopus arrhizus* | OP161823 |
| 7 | P-470 clinical | *Rhizopus arrhizus* | OP161824 |
| 8 | P-470 indoor | *Rhizopus arrhizus* | OP161825 |
| 9 | P-470 outdoor | *Rhizopus arrhizus* | OP161826 |
| 10 | P-564 clinical | *Rhizopus arrhizus* | OP161827 |
| 11 | P-564 indoor | *Rhizopus arrhizus* | OP161828 |
| 12 | P-564 outdoor | *Rhizopus arrhizus* | OP161829 |
| 13 | P-690 clinical | *Rhizopus arrhizus* | OP161830 |
| 14 | P-690 indoor | *Rhizopus arrhizus* | OP161831 |
| 15 | P-808 clinical | *Rhizopus delemar* | OP161832 |
| 16 | P-808 indoor(air) | *Rhizopus delemar* | OP161833 |
| 17 | P-808 indoor(swab) | *Rhizopus delemar* | OP161834 |
| 18 | P-808 outdoor | *Rhizopus delemar* | OP161835 |
| 19 | P-851 clinical | *Rhizopus microsporus* | OP161836 |
| 20 | P-851 indoor | *Rhizopus microsporus* | OP161837 |
| 21 | IL-2378 clinical | *Rhizopus homothalicus* | 710380 |
