# Supplementary figures and images for: Evaluation of environmental Mucorales contamination in and around the residence of COVID-19-associated mucormycosis patients
Source: Front Cell Infect Microbiol. 2022 Sep 2;12:953750. doi: 10.3389/fcimb.2022.953750 (PMC9478190; doi:10.3389/fcimb.2022.953750)

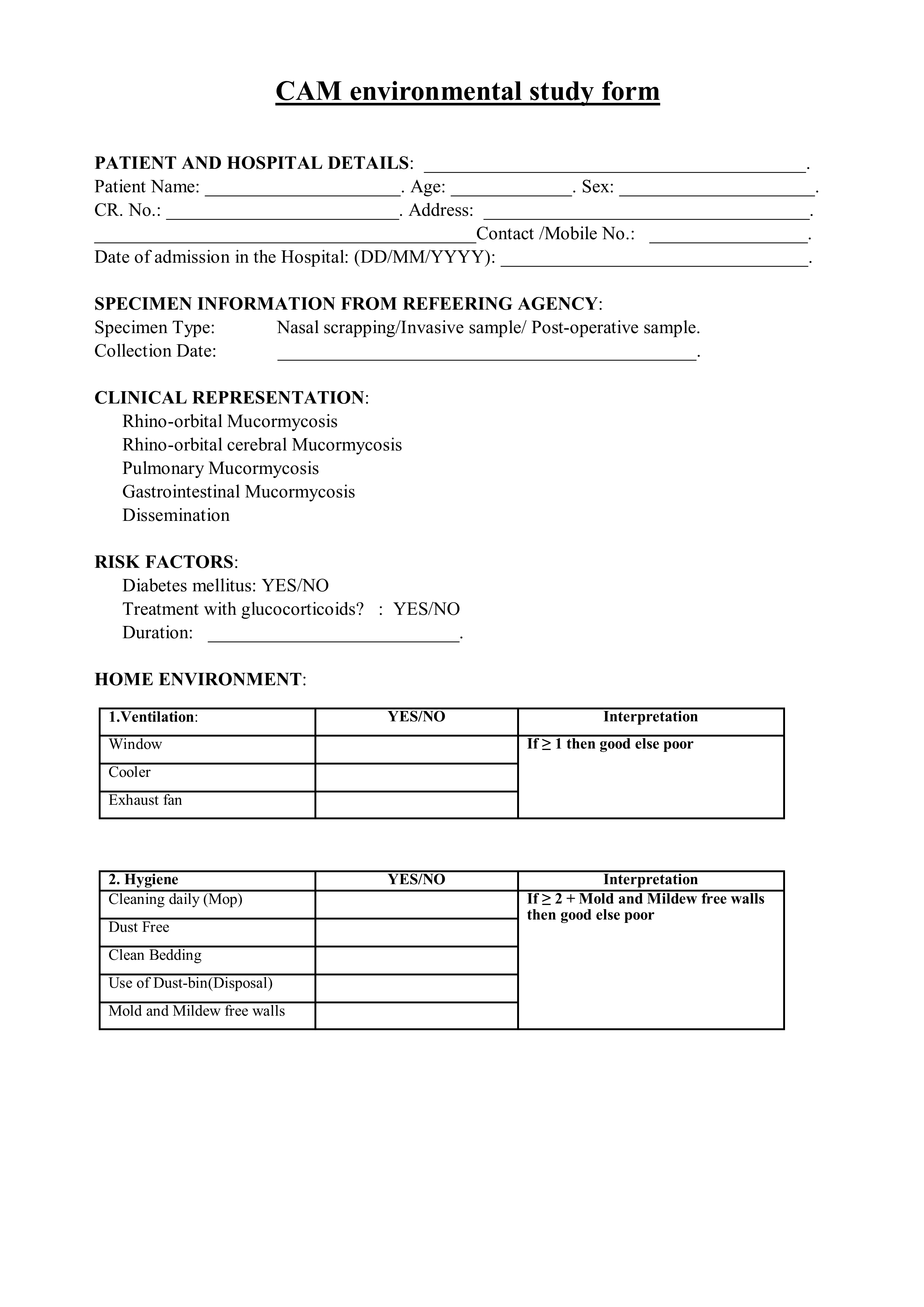

Supplement: Supplementary file 2 [file Image_1.tif]
